# Risks of suicide among family members of suicide victims: A nationwide sample of South Korea

**DOI:** 10.3389/fpsyt.2022.995834

**Published:** 2022-10-14

**Authors:** Jihoon Jang, Seong Yong Park, Yeon Yong Kim, Eun Ji Kim, Gusang Lee, Jihye Seo, Eun Jin Na, Jae-Young Park, Hong Jin Jeon

**Affiliations:** ^1^Department of Psychiatry, Depression Center, Samsung Medical Center, Sungkyunkwan University School of Medicine, Seoul, South Korea; ^2^Korea Psychological Autopsy Center, Seoul, South Korea; ^3^Department of Psychiatry, Keyo Hospital, Uiwang, South Korea; ^4^Department of Big Data Management, National Health Insurance Service, Wonju, South Korea; ^5^Department of Health Administration, Yonsei University Graduate School, Wonju, South Korea; ^6^Department of Psychiatry, Seoul Medical Center, Seoul, South Korea; ^7^Department of Health Sciences and Technology, Samsung Advanced Institute for Health Sciences and Technology, Sungkyunkwan University, Seoul, South Korea; ^8^Department of Medical Device Management and Research, Samsung Advanced Institute for Health Sciences and Technology, Sungkyunkwan University, Seoul, South Korea; ^9^Department of Clinical Research Design and Evaluation, Samsung Advanced Institute for Health Sciences and Technology, Sungkyunkwan University, Seoul, South Korea

**Keywords:** suicide survivor, risk of completed suicide, hazard model regression, South Korea, bereaved family

## Abstract

**Objective:**

Identifying the risks of completed suicide in suicide survivors is essential for policies supporting family members of suicide victims. We aimed to determine the suicide risk of suicide survivors and identify the number of suicides per 100,000 population of suicide survivors, bereaved families of traffic accident victims, and bereaved families with non-suicide deaths.

**Methods:**

This was a nationwide population-based cohort study in South Korea. The data were taken from the Korean National Health Insurance and Korea National Statistical Office between January 2008 and December 2017. The relationship between the decedent and the bereaved family was identified using the family database of the National Health Insurance Data. Age and gender were randomly matched 1:1 among 133,386 suicide deaths and non-suicide deaths. A proportional hazard model regression analysis was conducted after confirming the cumulative hazard using Kaplan–Meier curves to obtain the hazard ratio (HR) of completed suicide in suicide survivors.

**Results:**

Using 423,331 bereaved families of suicide victims and 420,978 bereaved families of non-suicide deaths as the control group, HR of completed suicide in suicidal survivors was found to be 2.755 [95% confidence limit (CL): 2.550–2.977]. HR for wives committing suicide after husbands' suicide was 5.096 (95% CL: 3.982–6.522), which was the highest HR among all relationships with suicide decedents. The average duration from suicide death to suicide of family members was 25.4 months. Among suicide survivors, the number of suicides per 100,000 people was 586, thrice that of people in bereaved families of traffic accident victims and in bereaved families of non-suicide deaths.

**Conclusion:**

The risk of completed suicide was three times higher in suicide survivors than in bereaved families with non-suicide deaths, and it was highest in wives of suicide decedents. Thus, socio-environmental interventions for suicidal survivors must be expanded.

## Introduction

Although the suicide rate is declining worldwide, about 800,000 people die each year by suicide. This is equivalent to 10.6 per 100,000 people (1). In Korea, which ranks first in suicide mortality rate among Organization for Economic Cooperation and Development (OECD) countries, the suicide rate has recently increased. In 2019, there were 26.9 suicide deaths per 100,000 people (a total of 13,799 deaths by suicide) (2). According to a recent meta analysis of 18 studies in six countries, the prevalence of exposure to suicide by family members and suicide by friends and colleagues during one's lifetime was 3.9 and 14.53%, respectively (3). One suicide is known to affect from six to 20 people on average, including extended family and coworkers (4, 5).

Family members of suicide victims are at increased risk for physical diseases such as pain, cardiovascular diseases, and cancer (6–9). They suffer significant complicated grief due to stigmatization, guilt, responsibility, shame, and rejection (10–16). Moreover, they experience symptoms of depression, anxiety, and post-traumatic stress disorder (PTSD) with an increased risk of psychiatric hospitalization (7, 17–25). Suicide survivors are at a higher risk for suicide compared to the general population. Their levels of suicide idea, suicide plans, and suicide attempts are 3, 5, 7, and 6 times higher, respectively, than those of the general population (26). Compared to bereavements in sudden deaths from natural causes, the risk of suicide attempts in suicide survivors is known to be 1.65 times higher (27).

There have been studies on completed suicide among family members of suicide victims. Suicide risk increased by 2.58 times in families with a history of completed suicide (28). According to a Swedish study (29), the probability of suicide was more than twice as high in first-degree relatives of 8,396 suicide deaths as in first-degree relatives of 7,568 non-suicide deaths (9.4 and 4.6%, respectively). In a large nested case-control study (30), the risk of a husband committing suicide was 46.2 when the spouse died by suicide, which was three times the risk when the spouse died from causes other than suicide. Parents who lost their children by suicide were twice as likely to commit suicide as those who lost their children through other means (risk: 2.54 and 1.4, respectively) (31). When a sibling committed suicide, the remaining sibling's suicide risk was 2.44 for men and 3.19 for women, which was more than twice that when a sibling died by other means (32). Meanwhile, in South Korea, a large-scale study on the actual completed suicide risk of suicide survivors has not yet been conducted.

To determine the risk of completed suicide among suicide survivors, this study carried out a large-scale analysis of the bereaved families of all suicide deaths in 10 years in South Korea. The hazard ratio (HR) for completed suicide among suicide survivors was explored for each family relationship by establishing bereaved families of non–suicide victims as control group. The average length of time it took to commit suicide was examined. Furthermore, suicides per 100,000 people and the proportion of completed suicides to the number of all deaths in the suicide survivors were compared to those in families with traffic accident deaths and families with deaths other than suicide including traffic accident deaths. The analysis considered surviving family relationships, and the gender of spouses, parents, children, and siblings of the suicide victims.

## Materials and methods

### Data

This population-based cohort study collected data for analysis from the National Health Insurance Service (NHIS) and Statistics Korea. The National Health Information database of the NHIS includes data such as deaths, medical institution treatments, and national examinations. It also provides the demographic and socioeconomic characteristics of all citizens. In this study, in addition to the generally provided national health information database, a family database, which could be accessed through policy research, was used to link family relationships between individuals for an in-depth analysis of suicide survivors. Statistics Korea's data on the causes of death are national statistics that provide a standardized cause classification based on the World Health Organization's International Classification of Diseases (ICD). List of people who died between January 2008 and December 2017 was extracted from NHIS data. After identifying the deceased among bereaved family through linking family data, it was sent to Statistics Korea. Through the microdata integrated service of Statistics Korea, the ICD code was linked to the list of deaths. This study was carried out after obtaining approval from the Institutional Review Board (IRB) of Samsung Medical Center.

### Study population

By linking data from the NHIS and Statistics Korea, a 1:1 randomized matching of gender and age was conducted for 133,386 suicide deaths from January 2008 to December 2017. Ages were matched in five groups: under 20, 20–39, 40–59, 60–79, and >80 years old. When linking the family database, cases in which there were two or more bereaved family members related to a spouse's father and mother due to remarriage and cases without bereaved families were removed. Thus, survival analysis was performed for survivors of 126,344 suicide deaths and 125,513 non-suicide deaths.

For bereaved families, the family database of the NHIS was linked. The scope of the bereaved family was limited to spouses, fathers, mothers, brothers, and sisters to increase the reliability of data. The number of suicide victims of suicide survivors was identified by the family relationships of 126,344 suicide victims, excluding those with no survivors or overlapping relationships. The number of suicides was examined by family relationships for 55,747 traffic accident fatalities (V01–V99) and 2,186,890 deaths other than by suicide.

### Outcome variable: Suicide

Statistics Korea's data on the cause of death were linked from 2008 to 2017 for the outcome variable of survival analysis as suicide of the bereaved family. According to the ICD, the code X60–X84 classified a death as suicide. Deaths not classified as suicide were defined as non-suicides.

### Covariates

Regarding the family of origin, age, gender, health insurance amounts, the national health insurance classification of the subscriber, divided into employee health insurance and local—subscriber health insurance were used as correction variables. Regarding the bereaved families of suicide victims and the bereaved families of non–suicide victims, only age and gender were used as correction variables, because only minimal linkage was possible according to the personal information policy.

### Statistical analysis

The duration from non-suicide deaths randomly matched with all suicide deaths to suicide in the bereaved family was investigated. If bereaved family members did not die by committing suicide, the observation period was set until December 2017. The cumulative hazard of completed suicide was examined for the family members of suicide victims and the control group using Kaplan–Meier curves. Since the suicide rate was not high in absolute numbers, the Y-axis was limited to 0.9–1.0. A log-rank test was performed to analyze statistically significant differences between the two curves. Through proportional hazard model regression analysis, the HR of completed suicide among suicide survivors was obtained. In addition, the average time was obtained by calculating the duration from suicide decedents to the suicide death of a family member. This analysis was conducted for suicide decedents and their spouses, fathers, mothers, children, and siblings. All data were analyzed using SAS version 9.4 (SAS Institute) in this study.

## Results

**Figure 1:**
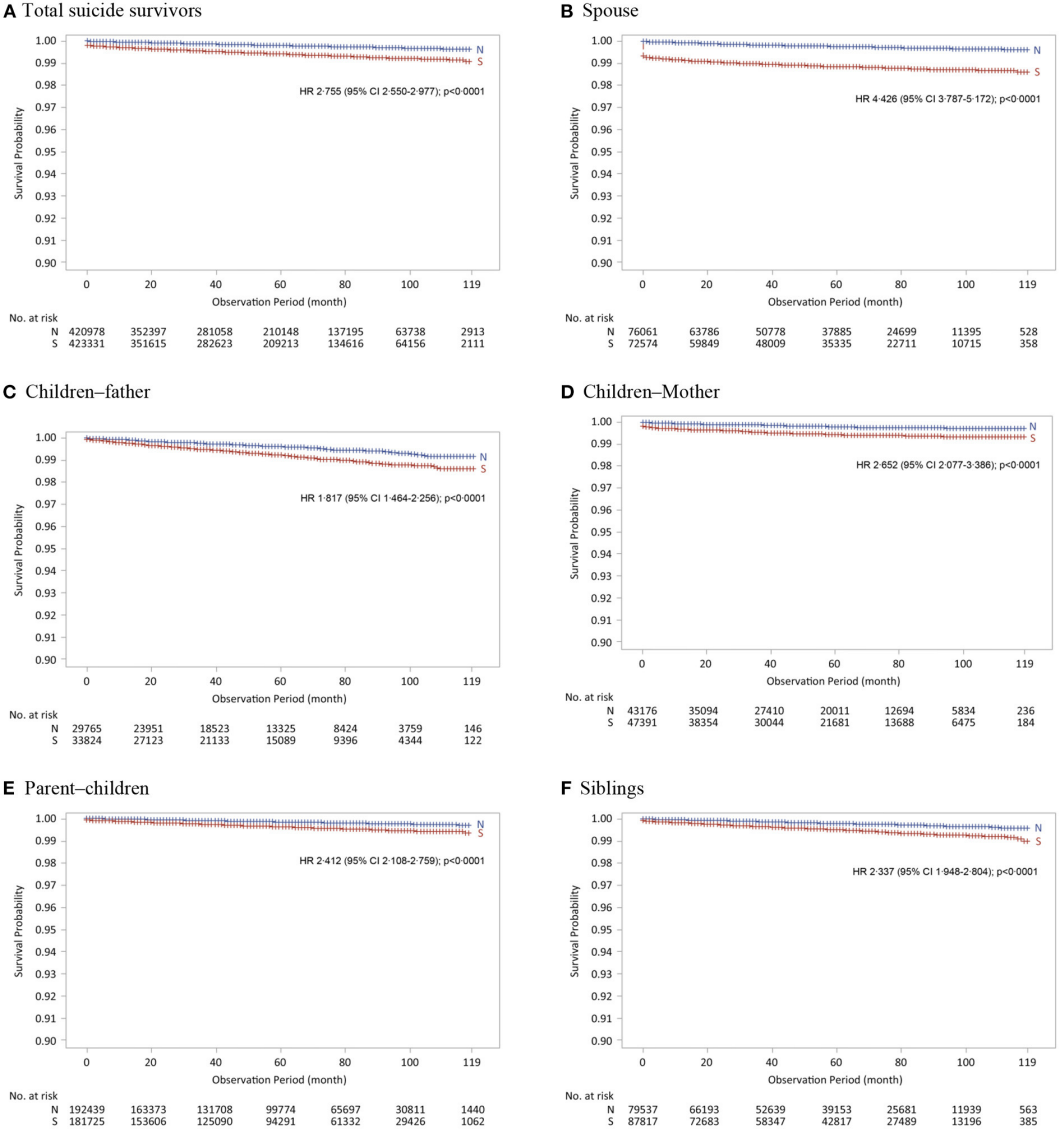
Kaplan–Meier cumulative hazard for suicide in suicide survivors and controls. **(A)** Total suicide survivors. **(B)** Spouse. **(C)** Children–father. **(D)** Children–Mother. **(E)** Parent–children. **(F)** Siblings. N, bereaved families of non–suicide deaths; S, bereaved families of suicide deaths.

Using 423,331 bereaved families of suicide deaths, along with 420,978 bereaved families of non-suicide deaths as the control group, the proportional hazard assumption was confirmed to be satisfied through Kaplan–Meier curves (Figure 1A). Moreover, the cumulative hazard was expressed through the Kaplan–Meier curve for each family member of suicide victims, including spouses, parents, children, and siblings (Figures 1B–F). Considering gender, the proportional hazard assumption was satisfied (Supplementary Figure 1). As a result of the log-rank test, the Kaplan–Meier curve between the two groups showed that the *P*-values for all family relationships were significantly <0.05.

Proportional hazard model regression analysis was performed according to family relationships to obtain the HR of completed suicide in family members using the bereaved families of non–suicide victims as the control group. The risk of completed suicide in all suicide survivors was found to be about three times higher than that in bereaved families of the non-suicide deaths (Table 1). In terms of family relationships, when a spouse committed suicide, suicide by the remaining spouse had the highest HR, followed by mothers committing suicide after the suicide of a child, the remaining children committing suicide after a parent's suicide, the remaining sibling committing suicide after a sibling's suicide, and fathers committing suicide after a child suicide.

**Table 1:** Hazard ratio (HR) of suicide in suicide survivors than in non-suicide survivors.

**Relationship with suicide decedents (suicide decedents -suicide survivor)**		**HR (95% Confidence Limits)**	***P*-value**	**Average duration from suicide victim's death to family member suicide (month)**
Spouse	Spouse (total)	4.426 (3.787–5.172)	<0.0001	13.5
	husband-wife	5.096 (3.982–6.522)	<0.0001	12.5
	wife-husband	3.871 (3.165–4.733)	<0.0001	14.2
Father	Father (total)	1.817 (1.464–2.256)	<0.0001	32.8
	Son-father	1.830 (1.401–2.391)	<0.0001	32.4
	Daughter-father	1.759 (1.214–2.548)	0.0029	33.6
Mother	Mother (total)	2.652 (2.077–3.386)	<0.0001	18.1
	Son-mother	2.489 (1.833–3.380)	<0.0001	19.7
	Daughter-mother	3.123 (2.067–4.719)	<0.0001	15.4
Children	Children (total)	2.412 (2.108–2.759)	<0.0001	32.3
	Father-son	2.119 (1.718–2.614)	<0.0001	36.7
	Father-daughter	1.664 (1.207–2.294)	0.0019	36.3
	Mother-son	2.712 (2.118–3.474)	<0.0001	29.1
	Mother-daughter	4.666 (3.059–7.118)	<0.0001	22.1
Siblings	Siblings (total)	2.337 (1.948–2.804)	<0.0001	33.5
	Brother-brother	1.822 (1.380–2.407)	<0.0001	35.8
	Brother-sister	2.396 (1.599–3.591)	<0.0001	35.8
	Sister-brother	2.782 (1.835–4.218)	<0.0001	35.6
	Sister-sister	3.312 (2.116–5.183)	<0.0001	24.7
Total suicide survivors		2.755 (2.550–2.977)	<0.0001	25.4

Regarding the risk of suicide in the spouses, parents, children, and siblings, taking gender into account, the HR for wives completing a suicide attempt when the husband died by suicide was the highest, followed by the HR for daughters committing suicide after a mother's completed suicide. When a sister died by suicide, the HR of other sisters committing suicide was higher than other gender-sibling relationships. When a daughter died by suicide, the mother's suicide HR was higher than the risk of a mother's completed suicide risk in the case of a son's suicide death. Furthermore, the father's risk of suicide when a son died by suicide was higher than the risk of a father's suicide when a daughter died by suicide. Thus, parents had a higher risk of suicide when their children of the same gender died by suicide.

The average duration from suicide decedents to suicide-by-suicide-survivors was found to be 25.4 months. In terms of detailed family relationships, the average duration from suicide of spouse to death of the remaining spouse by suicide was the shortest. The average duration from the suicide of a child to the suicide of the mother was found to be the second shortest, followed by the average duration from the suicide of a parent to the suicide of a child, the average duration from the suicide of a child to the suicide of the father, and the average duration from the suicide of the sibling to the suicide of the remaining sibling.

The number of suicides per 100,000 people among suicide survivors was found to be 586 after exclusion criteria were applied to the data (Table 2). During the 10-year period, the number of suicide deaths among the bereaved families of traffic accident victims was confirmed to be 216 per 100,000 people. In addition, among the bereaved family members of all deaths other than suicide, the number of suicide deaths was 207 per 100,000 people. The proportion of deaths by suicide among the deaths of the family members of suicide victims was 15.5%, which was about three times higher than that for suicide among the bereaved families of traffic accident victims and the bereaved families of those who died through causes other than suicide (Table 3).

**Table 2:** Suicide by family relationships among suicide survivors, bereaved families of traffic accident victims, and bereaved families of deaths other than by suicide.

	**Cause of death in decedents**											
	**Suicide**				**Traffic accident**				**All death other than suicide**			
**Relationship^a^**	**Decedent (*n*)**	**Bereaved family (*n*)**	**Suicides in bereaved family (*n*)**	**Suicides per 100,000 bereaved family (*n*)**	**Decedent (*n*)**	**Bereaved family (*n*)**	**Suicides in bereaved family (*n*)**	**Suicides per 100,000 bereaved family (*n*)**	**Decedent (*n*)**	**Bereaved family (*n*)**	**Suicides in bereaved family (*n*)**	**Suicides per 100,000 bereaved family (*n*)**
Husband-wife	53,890	53,890	375	696	27,248	27,248	46	169	925,035	925,035	1,347	146
Wife-husband	18,684	18,684	459	2,457	6,575	6,575	41	624	259,431	259,431	2,016	777
Son-father	22,210	22,210	171	770	10,689	10,689	47	440	68,789	68,789	387	563
Daughter-father	11,614	11,614	90	775	2,217	2,217	12	541	27,298	27,298	157	575
Son-mother	32,710	32,710	158	483	15,278	15,278	31	203	142,891	142,891	399	279
Daughter-mother	14,681	14,681	99	674	2,630	2,630	4	152	39,479	39,479	128	324
Father-son	49,284	70,632	262	371	25,247	38,158	72	189	972,377	1,499,594	2,862	191
Father-daughter	38,491	55,560	98	176	18,994	28,684	40	139	637,730	960,197	836	87
Mother-son	23,451	33,588	223	664	10,831	16,923	55	325	833,538	1,246,773	3,199	257
Mother-daughter	15,805	21,945	113	515	6,928	10,489	12	114	396,154	550,730	570	103
Brother-brother	23,760	30,422	154	506	10,393	1*3,237	29	219	109,756	140,416	346	246
Brother-sister	20,060	27,273	89	326	8,766	11,729	10	85	76,929	99,403	105	106
Sister-brother	12,562	15,588	96	616	2,146	2,694	3	111	36,933	47,372	101	213
Sister-sister	10,138	14,534	94	647	1,786	2,449	6	245	31,891	43,480	60	138
Total	126,344	423,331	2,481	**586**	55,747	189,000	408	**216**	2,186,890	6,050,888	12,513	**207**

a Relationship to the previous decedent (previous decedent-bereaved family).

**Table 3:** Ratio of suicide deaths among bereaved family deaths by family relationship.

	**Cause of death in decedents**								
	**Suicide**			**Traffic accident**			**All death other than suicide**		
**Relationship^a^**	**(a) Non-suicide death in bereaved family (*n*)**	**(b) Suicide in bereaved family (*n*)**	b/(a+b)*100 (%)	**(a) Non-suicide death in bereaved family (*n*)**	**(b) Suicide in bereaved family (*n*)**	b/(a+b)*100 (%)	**(a) Non-suicide death in bereaved family (*n*)**	**(b) Suicide in bereaved family (*n*)**	b/(a+b)*100 (%)
Husband-wife	2,217	375	14.5	1,311	46	3.4	73,312	1,347	1.8
Wife-husband	1,610	459	22.2	1,261	41	3.1	56,411	2,016	3.5
Son-father	2,717	171	5.9	1,183	47	3.8	11,873	387	3.2
Daughter-father	973	90	8.5	183	12	6.2	2,744	157	5.4
Son-mother	3,075	158	4.9	1,485	31	2.0	26,621	399	1.5
Daughter-mother	665	99	13.0	161	4	2.4	3,139	128	3.9
Father-son	608	262	30.1	292	72	19.8	15,872	2,862	15.3
Father-daughter	176	98	35.8	77	40	34.2	3,156	836	20.9
Mother-son	561	223	28.4	339	55	14.0	34,431	3,199	8.5
Mother-daughter	140	113	44.7	98	12	10.9	3,777	570	13.1
Brother-brother	455	154	25.3	223	29	11.5	4,328	346	7.4
Brother-sister	128	89	41.0	67	10	13.0	1,019	105	9.3
Sister-brother	150	96	39.0	40	3	7.0	1,038	101	8.9
Sister-sister	60	94	61.0	46	6	11.5	798	60	7.0
Total	13,535	2,481	**15.5**	6,766	408	**5.7**	238,519	12,513	**5.0**

a Relationship to the previous decedent (previous decedent-bereaved family).

## Discussion

The risk of completed suicide among suicide survivors was found to be about three times higher than that among the bereaved families of non–suicide deaths. The average duration from a suicide death to the suicide of family members was about 2 years. In terms of family relationships, the risk was the highest when the family member of a suicide victim was a spouse, followed by the mother, sibling, child, and father. When gender was taken into account in the bereaved relationship, the HR of completed suicide was higher in family members of the same gender. Furthermore, the number of suicides per 100,000 population among suicide survivors was about three times the number of deaths per 100,000 people due to suicide among the bereaved families of traffic accidents and the bereaved families of non-suicide deaths. These results are consistent with previous studies (28, 29) showing that suicide survivors had a high risk of suicide. The results of a nested case-control study (28) in Denmark also showed that the risk of death by suicide was three times higher in the presence of a first-degree completed suicide. Similarly, a large-scale Swedish study (29) found that the suicide rate was twice as high for 33,173 suicide survivors than for 28,945 bereaved families of those who died from causes other than suicide.

The results of this study are also consistent with previous studies (30–34) showing that suicide risk was high in the spouses, parents, and siblings of suicide victims. According to a nested case-control Danish study (33), the risk of suicide in the remaining spouse of someone who died by suicide and the death of a spouse due to other causes was 21.69 and 7.65, respectively. In an additional study (30), according to the gender of the remaining spouse, the suicide risk of a husband who lost his wife due to suicide was 4.6 times higher compared to losses due to other reasons. Moreover, the suicide risk of a wife who lost her husband due to suicide was about 4.8 times higher compared to a loss due to other causes. When a child was lost by suicide, the parents' suicide risk was 1.8 times higher than if the loss was due to other causes (31). These results are consistent with those in this study.

People bereaved by a suicide death feel significantly more stigma, shame, responsibility, and guilt than those bereaved by other unnatural sudden deaths (10, 35). In this study, the number of suicide victims per 100,000 population was three times higher in suicide survivors than in families bereaved due to abrupt and unexpected traffic accidents. Similarly, previous studies found that suicide risk was increased in first-degree relatives who had lost a family member by sudden death. Among them, those who lost a family member by suicide had the highest risk of suicide (36). In addition, it is consistent with the results of a recent study (37) that among sudden bereavements, a higher level of perceived stigma was associated with the level of suicide attempt risk, which is 2.7 times higher.

This study was consistent with previous studies showing that the risk of suicide was higher than that of men when the gender of the survivors was women. However, for children who lost their parents by suicide, children of the same gender as their deceased parents had a higher risk of suicide. According to a previous study (34), the risk of suicide for a husband was 3.6 when he lost his wife by suicide, while the risk of suicide for a wife was 4.5 when a husband committed suicide, indicating that the risk of suicide for a wife was higher. The suicide risks of mothers and fathers after losing a child by suicide were 3.47 and 1.90, respectively, implying that mothers were more likely to be affected by suicide (31). These results are consistent with those of the current study. A Swedish study (32) found that the risks of suicide in siblings leading to the suicide of the remaining sibling were 2.44 and 3.15 for males and females, respectively. Women may be more affected by the loss of a family member than men because women value family support and close relationships more than men (38).

Suicide familial aggregation can be explained by genetic and non-genetic factors (39). First, there is a genetic aspect to the cause of such high suicide risk of suicide survivors (40–42). According to a review (41), completed suicide was higher in monozygotic twins than in dizygotic twins. Thus, the genetic contribution to suicide is high. Recently, genotypes related to suicide have been revealed (42). According to a recent cohort study in Sweden, the heritability of completed suicide was found to be slightly higher in women than in men (40). This supports the findings of this study that women were more affected by family suicide. In addition to biological factors, familial factors such as disorganization, breakup, and intrafamily violence can increase the risk of suicide. Accordingly, family members of suicide victims who share a dysfunctional familial history might be more vulnerable to suicide (43). In this study, the risk of suicide was the highest for spouses without genetic commonality among all family relationships. The death of a spouse is the highest stressor in life (44). It seems to have a greater impact than the death of other family members.

In this study, the average period from the suicide of a family member to the suicide of a bereaved family member was 25.4 months. The average duration to the suicide of a mother who lost a spouse or child was relatively short, at < 2 years, and it was about 3 years in children, siblings, and fathers. According to previous studies (45, 46), the turning point at which the grief of suicide loss integrates is about 3–5 years, and the duration for suicide survivors to recover from psychiatric symptoms is 3 years. Moreover, the odd ratio of suicide in mothers who lost their children by suicide was the highest at 76.05 during the first month, then decreased to about 4.5 from 1 to 12 month, and remained at around 1.8 after 1 year (31). Active psychiatric treatment intervention and support are needed since the risk of suicide is high for 2–3 years from the time a family member commits suicide.

This study had several limitations. First, when linking data for survival analysis, only age and gender were used as correction variables for suicide survivors due to personal information privacy. Second, the exact date of death could not be confirmed when linking data from the NHIS and Statistics Korea. Thus, cases with the same year and month of death were excluded from the survival analysis. Third, this study was carried out using a Korean population database. Therefore, generalization of the study results to other ethnicities should be done with caution. Fourth, sequelae of intentional self-harm (ICD code Y87.0) and undetermined death (codes Y10–Y32, Y87.2) were classified as non-suicides, not as suicide. Undetermined deaths are not negligible proportion of external deaths in South Korea, therefore suicide may have been underestimated and non-suicides overestimated (47, 48). In future studies, sensitivity analysis having as outcomes undetermined deaths (UD) and the ratio UD/suicide should be performed. Despite these limitations, this study was significant in that it examined the actual risk of suicide death in the spouses, parents, children, and siblings, considering gender by targeting the bereaved families of all suicide victims up to 10 years in Korea. It also identified the period during which the suicide survivors were especially vulnerable to suicide.

In conclusion, the suicide risk of survivors of those who died by suicide over the past 10 years in Korea was confirmed to be about three times higher than the suicide risk of bereaved families of those who died from causes other than suicide. In particular, the risk of suicide for a wife after the suicide of her husband was the highest at 5 times, followed by the suicide risk for a daughter after a mother's suicide at 4.7 times. The number of suicides per 100,000 family members of suicide victims was three times the number of suicides per 100,000 bereaved family members of traffic accident fatalities or the bereaved families of deaths due to causes other than suicide. Furthermore, the average duration of committing suicide for suicide survivors was 2 years. As such, intensive psychiatric treatment intervention and support are needed for the family members of suicide victims in the early stages of loss who are vulnerable to suicide.

## Data availability statement

The raw data supporting the conclusions of this article will be made available by the authors, without undue reservation.

## Ethics statement

The studies involving human participants were reviewed and approved by Institutional Review Board (IRB) of Samsung Medical Center. Written informed consent from the participants' legal guardian/next of kin was not required to participate in this study in accordance with the national legislation and the institutional requirements.

## Author contributions

JJ participated in the study design, conceptualization, data interpretation, wrote the first manuscript drafting, and revised new drafts from co-authors. SP, YK, and HJ participated in the study design, data curation, formal analysis, and wrote the manuscript. EK and EN participated in conceptualization, manuscript drafting, and revising of the manuscript. GL, J-YP, and JS participated in the project administration and data presentation. HJ participated in study design and conception, data curation, and manuscript drafting. All authors read and approved the final manuscript.

## Funding

This research was supported by the Development of screening tools for high suicide risk group and evaluation tools of severity of suicide risk, and validation of their effectiveness (HL19C0001) funded by the Ministry of Health and Welfare, by the Healthcare AI Convergence R&D Program through the National IT Industry Promotion Agency of Korea (NIPA) funded by the Ministry of Science and ICT (No. S0254-22-1001), and by a grant of the Korea Health Technology R&D Project through the Korea Health Industry Development Institute (KHIDI), funded by the Ministry of Health & Welfare, Republic of Korea (No. HR21C0885). This study used NHIS-NHID data (NHIS-2020-1-530) gathered by Korea's National Health Insurance Service (NHIS). This study was mainly supported by the Interagency Committees of the Korean National Government (Suicide Prevention Action Plans in 2018: 1-1 the Korean National Investigations of 70,000 Suicide Victims Through Police Records).

## Conflict of interest

The authors declare that the research was conducted in the absence of any commercial or financial relationships that could be construed as a potential conflict of interest.

## Publisher's note

All claims expressed in this article are solely those of the authors and do not necessarily represent those of their affiliated organizations, or those of the publisher, the editors and the reviewers. Any product that may be evaluated in this article, or claim that may be made by its manufacturer, is not guaranteed or endorsed by the publisher.
