# Supplementary material for: Risks of suicide among family members of suicide victims: A nationwide sample of South Korea
Source: Front Psychiatry. 2022 Oct 14;13:995834. doi: 10.3389/fpsyt.2022.995834 (PMC9614235; doi:10.3389/fpsyt.2022.995834)
Supplement: Supplementary file 1 [file Table_1.DOCX]

Supplementary Figure 1. Kaplan–Meier cumulative hazard for suicide in suicide survivors and controls.

| A. Husband–wife  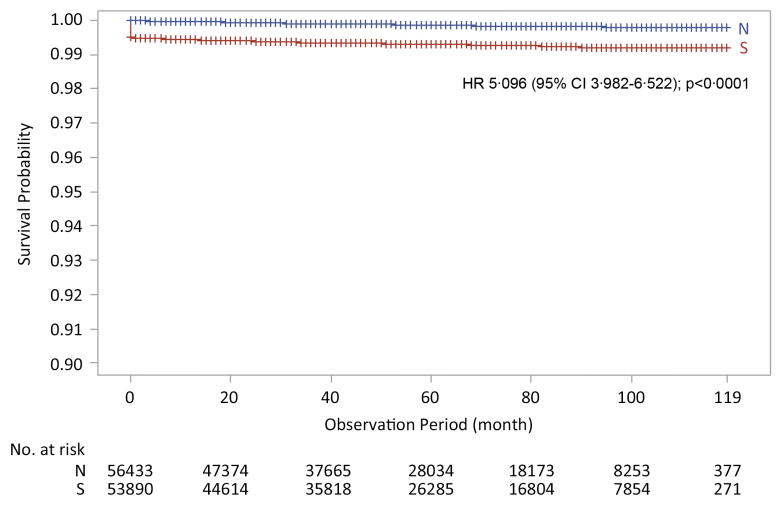 | B. Wife–husband  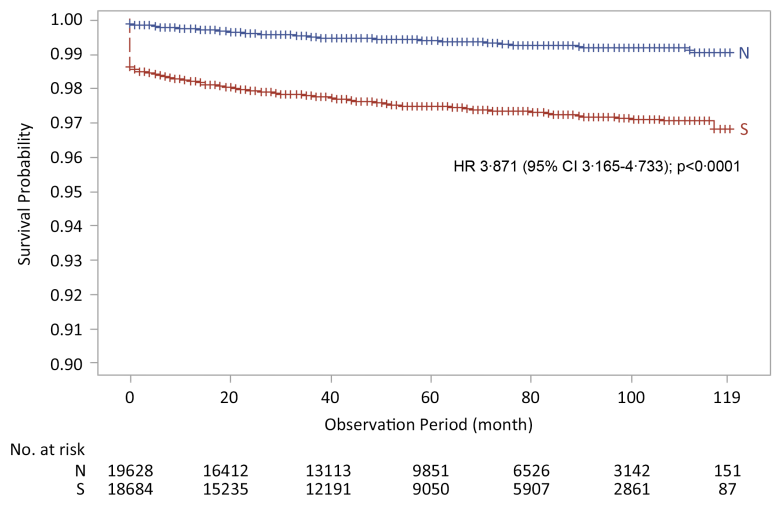 |
| --- | --- |
| C. Son–father  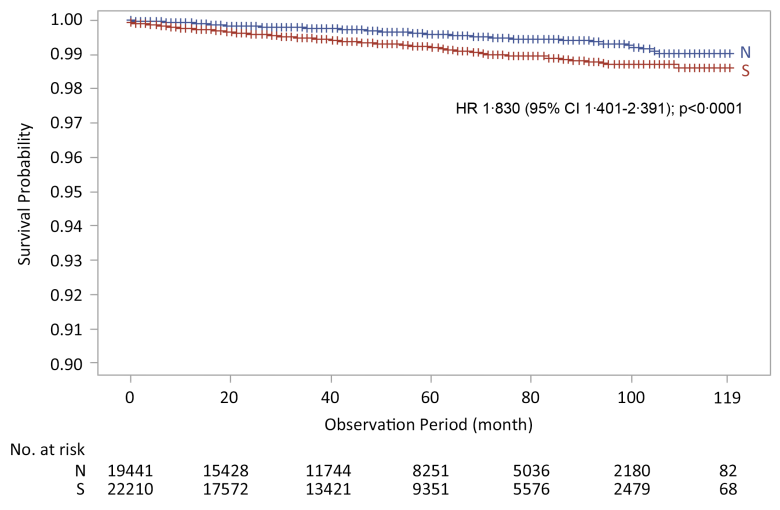 | D. Daughter–father  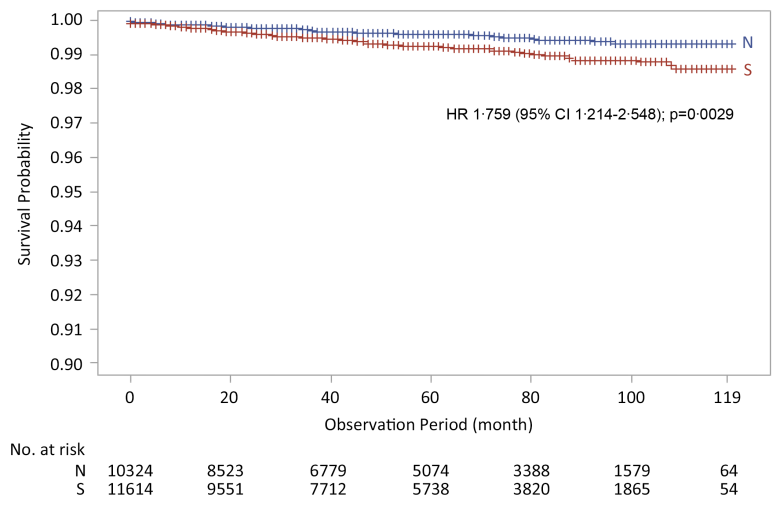 |
| E. Son–mother  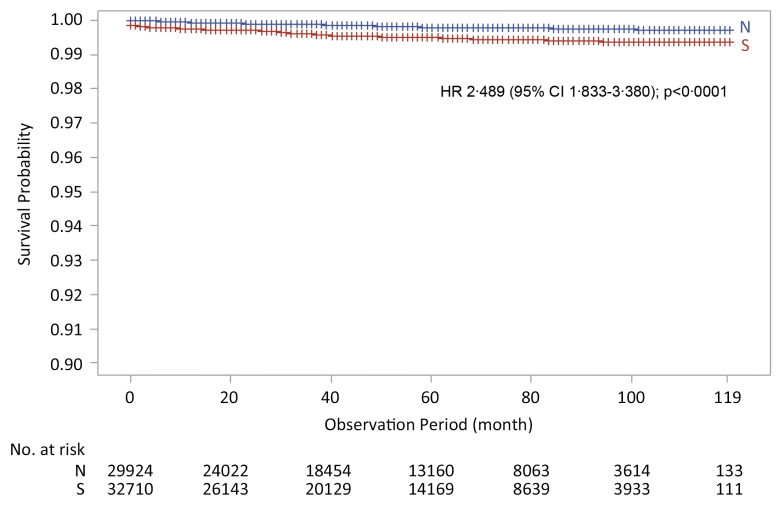 | F. Daughter–mother  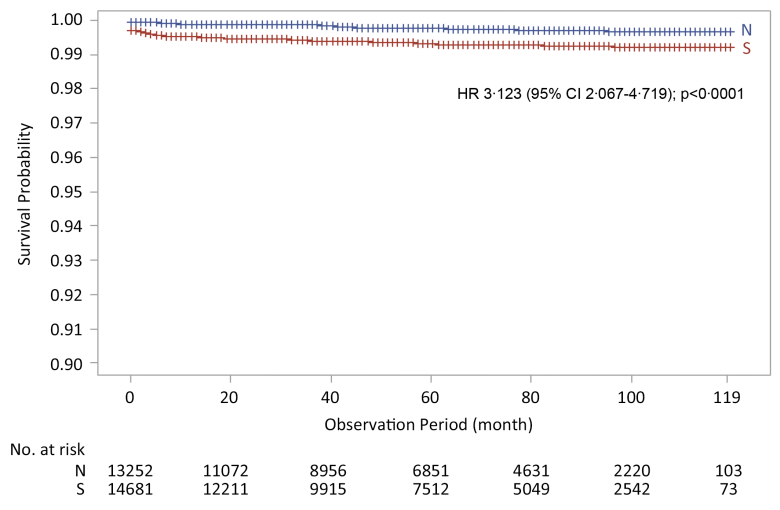 |
| G. Father–son  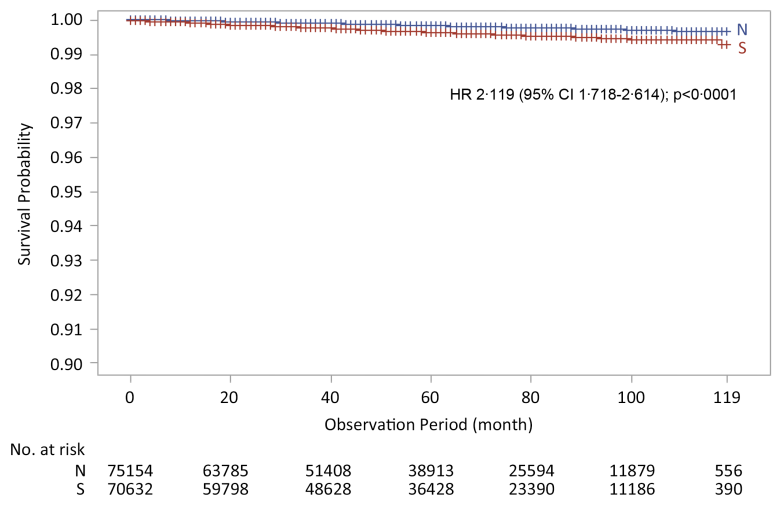 | H. Father–daughter  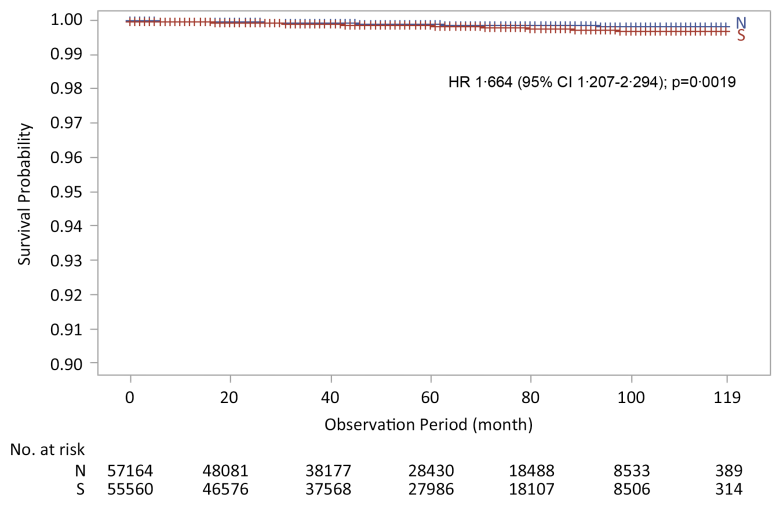 |
| I. Mother–son  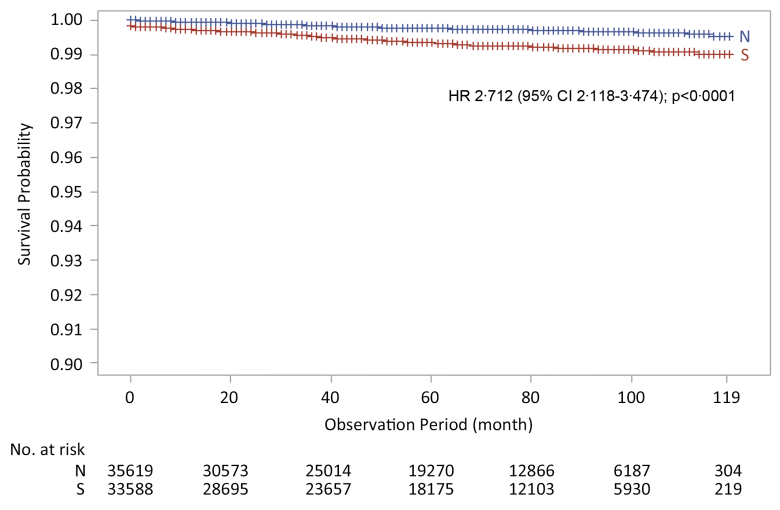 | J. Mother–daughter  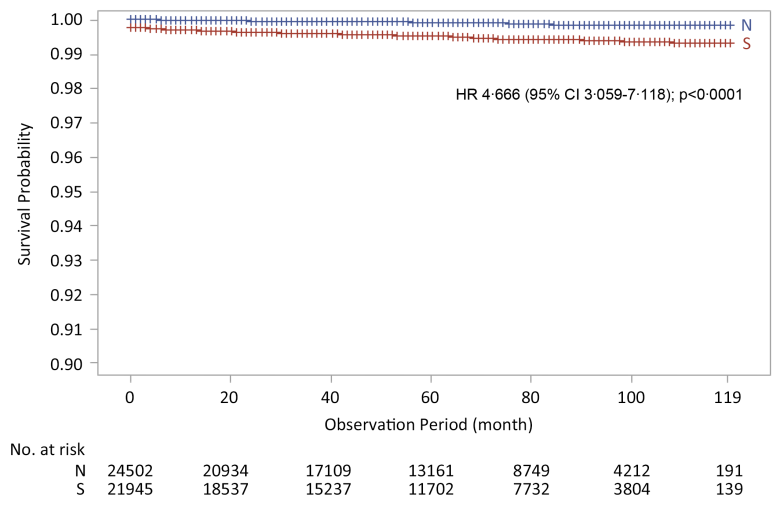 |
| K. Brother–brother  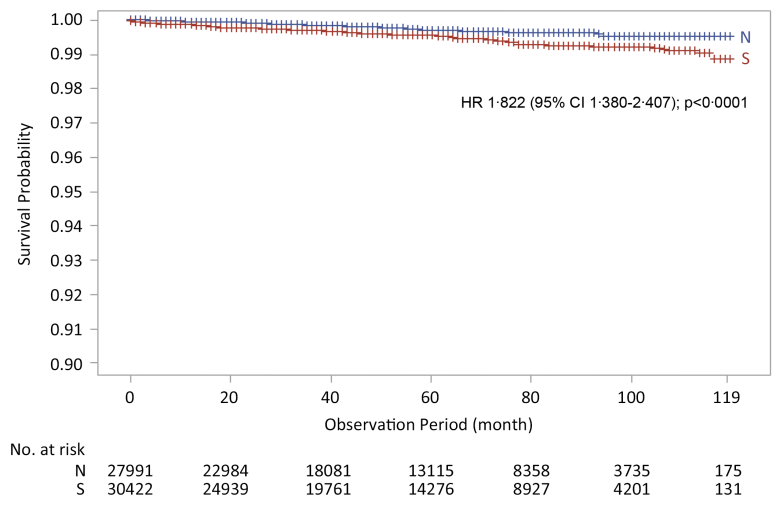 | L. Brother–sister  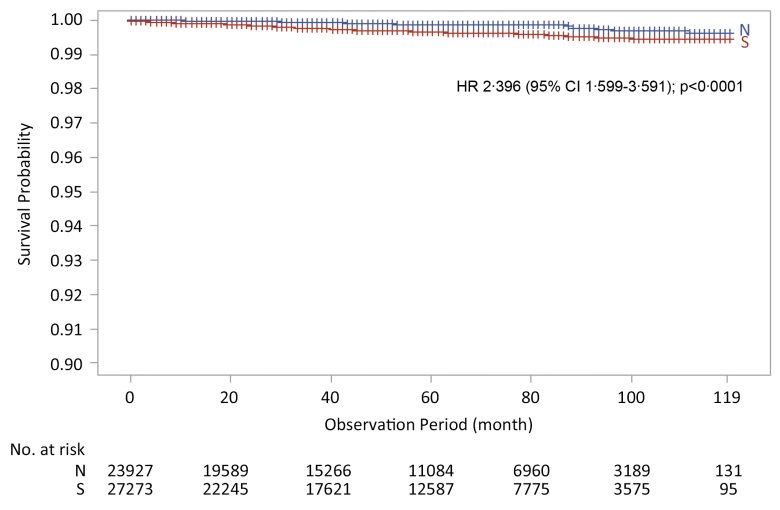 |
| M. Sister–brother  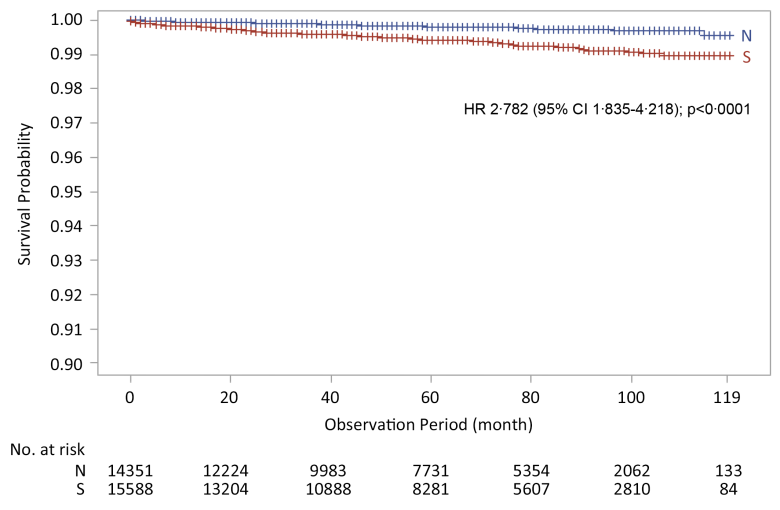 | N. Sister–sister  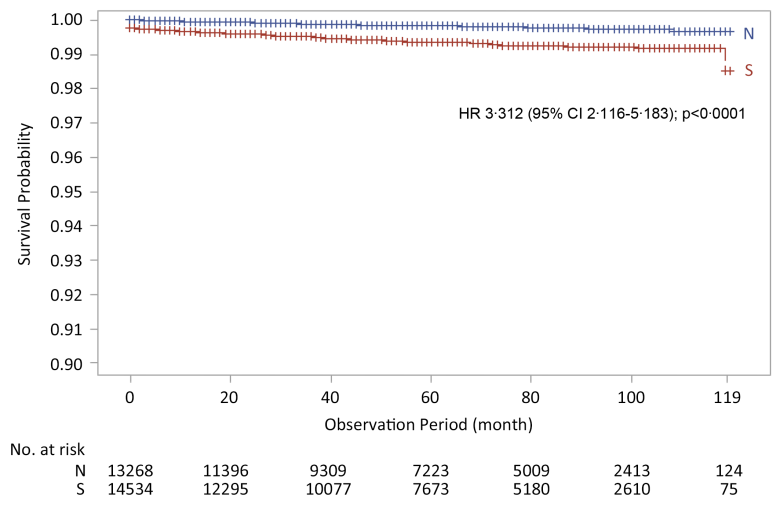 |

N: bereaved families of non–suicide deaths , S: bereaved families of suicide deaths
